# Lethal and Sublethal Effects of Carlina Oxide and *Acmella oleracea* Extract Enriched in *N*-Alkylamides on *Aculops lycopersici* (Acari: Eriophyidae) and Its Predator *Typhlodromus exhilaratus* (Acari: Phytoseiidae) in Laboratory Tests

**DOI:** 10.3390/insects16090879

**Published:** 2025-08-24

**Authors:** Thomas Giordano, Giuliano Cerasa, Ilaria Marotta, Mauro Conte, Ernesto Ragusa, Simona Tortorici, Gabriella Lo Verde, Filippo Maggi, Riccardo Petrelli, Marta Ferrati, Eleonora Spinozzi, Luigi Botta, Roberto Rizzo, Haralabos Tsolakis

**Affiliations:** 1 Department of Agricultural, Food and Forest Sciences, University of Palermo, Viale delle Scienze, Bldg. 5, 90128 Palermo, Italy; thomas.giordano@unipa.it (T.G.); giuliano.cerasa@unipa.it (G.C.); ilaria.marotta02@unipa.it (I.M.); mauro.conte@unipa.it (M.C.); ernesto.ragusa@unipa.it (E.R.); gabriella.loverde@unipa.it (G.L.V.); 2 CREA-Research Centre for Plant Protection and Certification, Viale Michelangelo, 1542, 90145 Palermo, Italy; simona.tortorici@crea.gov.it (S.T.); roberto.rizzo@crea.gov.it (R.R.); 3 Chemistry Interdisciplinary Project (ChIP) Research Center, School of Pharmacy, University of Camerino, Via Madonna delle Carceri, 62032 Camerino, Italy; filippo.maggi@unicam.it (F.M.); riccardo.petrelli@unicam.it (R.P.); marta.ferrati@unicam.it (M.F.); eleonora.spinozzi@unicam.it (E.S.); 4 Department of Engineering, Viale delle Scienze Ed. 8, 90128 Palermo, Italy; luigi.botta@unipa.it

**Keywords:** botanical pesticides, tomato russet mite, eriophyids control, side effects

## Abstract

The tomato russet mite, *Aculops lycopersici,* is considered one of the most important crop pests globally. The main control strategy is based on synthetic acaricides; however, they create resistant strains and ecological risks. In this context, biopesticides could be a viable and sustainable alternative for eriophyid control. In the present study, the toxic effects of a *N*-alkylamides-enriched extract obtained from *Acmella oleracea* and of carlina oxide, the main bioactive component of *Carlina acaulis*, on *A. lycopersici* were evaluated, as well as their side effects on the phytoseiid *Typhlodromus exhilaratus* under laboratory conditions. Six concentrations were tested for each product against *A. lycopersici* adults (0, 320, 640, 1280, 2500, and 5000 μL L^−1^), and the median concentration (1280 μL L^−1^) was evaluated against eggs and females of the phytoseiid *T. exhilaratus*. Both the *N*-alkylamides-enriched extract and carlina oxide showed total lethal effects (100% of mortality) towards *A. lycopersici* at the two highest concentrations. Moderate-to-high mortality was also recorded with the lower concentrations: from 42.22 to 97.78%. Probit analysis identified LC_50_ values of 205.32 μL L^−1^ for carlina oxide and 253.79 μL L^−1^ for the *N*-alkylamides-enriched extract, respectively. Carlina oxide showed a moderate ovicidal effect on *T. exhilaratus* eggs (50.00% hatching rate) on *T. exhilaratus*, and caused 39.13% mortality on females, whereas the *N*-alkylamides-enriched extract was less toxic, with a hatching rate of 88.00% and a mortality rate of 18.75% on females. In conclusion, carlina oxide and the *N*-alkylamides-enriched extract showed high toxicity on *A. lycopersici*, with a reduced effect on phytoseiid. These results highlight the potential of these products as sustainable means for the management of tomato russet mite.

## Simple Summary

The tomato russet mite (*Aculops lycopersici*) is a major pest of tomato crops worldwide. Current control methods rely on synthetic acaricides, which raise concerns due to resistance development and environmental impact. This study evaluated the acaricidal activity of two plant-derived products in laboratory tests: carlina oxide, the main compound of *Carlina acaulis*, and an extract enriched in *N*-alkylamides from *Acmella oleracea*. Both products showed high toxicity against eriophyids, with complete mortality observed at the highest concentrations tested. Carlina oxide was slightly more toxic and faster-acting, while the *N*-alkylamides extract also achieved full mortality within 72 h. Side effects on the predatory mite *Typhlodromus exhilaratus* revealed moderate toxicity for carlina oxide, particularly on eggs, whereas the *N*-alkylamides extract had limited impact, suggesting greater selectivity. These findings represent the first report of acaricidal activity of these specific compounds against *A. lycopersici* and indicate their potential as natural alternatives to synthetic pesticides. While this is promising for integrated pest management, further studies are needed to optimize formulations, assess field efficacy, and better understand the mode of action and safety profile of these substances.

## 1. Introduction

The rising global prominence of eriophyoid mites underscores their growing threat to sustainable agriculture. As these pests continue to expand their geographic range and their economic significance intensifies, there is an urgent need to deepen knowledge of their biology and management strategies [1]. Among the most damaging eriophyoid species, the tomato russet mite, *Aculops lycopersici* (Tryon) (Acari: Eriophyoidea), stands out as a major pest of Solanaceae crops, particularly tomatoes, with a widespread presence in tropical and temperate regions [2,3].

This mite feeds on the epidermal cells of leaves and stems, causing the characteristic bronzing and russeting that signals compromised plant health [4,5,6,7]. Such feeding behavior critically impairs the plant’s photosynthetic capacity, leading to broad leaf necrosis and, in severe infestations, to death of plants [8]. Under optimal conditions (26.5 °C and 30% relative humidity, RH) the eriophyid mite completes its life cycle in 6–7 days; females lay about 50 eggs in 20–30 days, mainly near veins or trichomes [9,10,11]. The eriophyid demonstrates rapid population growth, showing an increasing rate of natural increase by 0.267 and an ability to double its population in three days at 26 °C [1,12].

The management of *A. lycopersici* infestations traditionally relies on synthetic acaricides due to their proven effectiveness in controlling phytophagous mite populations. However, this underscores the pressing need for integrated pest management (IPM) strategies able to mitigate the ecological consequences of synthetic chemicals and address the escalating risk of acaricide resistance. These growing concerns have catalyzed significant interest in low-impact alternatives, particularly botanical pesticides, which are increasingly recognized for their cost-effectiveness and environmentally low-impact nature. Unlike synthetic chemicals, botanical pesticides typically exhibit lower environmental persistence, reduced toxicity to non-target organisms, minimal impact on human health, and a supportive role for natural pest enemies within the IPM framework [13].

Historically widespread in the early 20th century, the use of botanical pesticides declined during the mid-1900s with the rise of synthetic chemical alternatives [14,15,16,17,18,19,20,21,22,23,24]. It is noteworthy that natural products such as essential oils (EOs), extracts and compounds from plants belonging to various botanical families, such as Lamiaceae, Amaryllidaceae, and Myrtaceae, have been studied for their acaricidal activity against eriophyid mites in the last two decades [25,26,27,28,29]. In recent years, secondary metabolites extracted from the Asteraceae species have also gained increasing attention due to their strong biocidal and repellent effects against a range of arthropod pests [24,30,31,32]. However, studies on the effects of single compounds, EOs, or extracts are limited and mainly concern insects or mites that are not of agricultural interest [33]. Among plant-derived products, carlina oxide from *Carlina acaulis* L. (Asteraceae) and the *N*-alkylamides-enriched extract from *Acmella oleracea* (L.) R.K. Jansen (Asteraceae) have shown promising results in arthropod control, due to their insecticidal and acaricidal activities [24,25,34,35,36,37]. These products are effective in reducing egg-laying and longevity in both juvenile and adult stages of pest populations [24,35].

No studies to date have assessed the acaricidal activity of carlina oxide or the *N*-alkylamides-enriched extract on *A. lycopersici*, nor their potential side effects on its natural predator *Typhlodromus exhilaratus* Ragusa, 1977 (Acari: Phytoseiidae). The latter phytoseiid is widespread in the Mediterranean area [38] and studies in progress showed this predator as an important biocontrol agent of the tomato russet mite (Tsolakis et al., manuscript in preparation).

Given the ease of extraction of these plant-based acaricides and the ongoing need to identify additional solutions for controlling tomato russet mites, this study aimed to assess the lethal and sublethal effects of carlina oxide and the *N*-alkylamides-enriched extract on *A. lycopersici* under laboratory conditions. Additionally, we simultaneously evaluate their side effects on *T. exhilaratus* to focus on the broader ecological implications of these botanicals.

## 2. Materials and Methods

### 2.1. Extraction and Analysis of Carlina Oxide and N-Alkylamides-Enriched Extract

Carlina oxide was extracted through hydrodistillation from *C. acaulis* dry roots (Minardi & Figli S.r.l., Bagnacavallo, Ravenna, Italy; batch no C-230223-3) following the procedure previously reported by Rizzo et al. [24]. The compound was obtained with a yield of 0.87% (*w*/*w*), and its structure and purity were confirmed by GC-MS and NMR analyses and were consistent with those reported in the literature [34]. After hydrodistillation, carlina oxide was stored at −20 °C until biological assays. As regards the *N*-alkylamides-enriched extract, it was obtained from dry inflorescences of *A. oleracea* cultivated and collected in 2022 by Dr. Ettore Drenaggi (Castelfidardo, Italy, 43°27′16” N; 13°31′52” E, 38 m a.s.l.). Before extraction, the particle size of the inflorescences was reduced to 1.5 mm using a plant shredder (Albrigi, mod. E0585 purchased from Stallavena, Verona, Italy). The *N*-alkylamides-enriched extract was prepared from a supercritical fluid extract previously obtained with TH22–10 ×2 supercritical CO_2_ extraction equipment (Toption Instrument Co. Ltd., YanTa District, Xi’an, China) [37]. In detail, the above-mentioned extract was processed in a wiped film short-path molecular distillator (VKL 70–5 FDRR-SKR-T, VTA Verfahrenstechnische Anlagen GmbH & Co. KG, Niederwinkling, Germany) following the methodology previously reported [37]. Then, the quantification of the main *N*-alkylamides was performed by high-performance liquid chromatography with diode array detection (HPLC-DAD) as previously reported by Ferrati et al. [37]. Both products used in the present study were in liquid state.

### 2.2. Rearing of Aculops lycopersici

The tomato russet mite was reared on *Solanum nigrum* L. (Solanaceae) plants under controlled conditions. Berries of *S. nigrum* were collected in October 2023 from field at the Department of Agricultural, Food and Forest Sciences (SAAF), University of Palermo, Italy (38°6′25.03″ N, 13°21′0.19″ E). After collection, the fruits were dissected to extract the seeds, which were then placed on filter paper at room temperature for three days to dry. The seeds were subsequently stored in a glass container and maintained for six months in a climate-controlled chamber set at 9 ± 1 °C, 35 ± 5% RH, and constant darkness (0:24 h light:dark (L:D), photoperiod).

In the spring of 2024, the seeds were sown in groups of approximately 15 per pot, using plastic containers (22 × 22 × 26 cm). The substrate consisted of peat, topsoil (Gramoflor^®^, GmbH and Co. KG, Vechta, Germany), and expanded vermiculite (Gebearth^®^, RaAmon srl, Roma, Italy). A number of the germinated plants were used for *A. lycopersici* rearing, while the remaining plants were used for leaf sampling to prepare the experimental units (EU).

Many individuals of *A. lycopersici* were collected in May 2024 from naturally occurring *S. nigrum* plants in field at Balestrate (Palermo, Italy) (38°1′32.84″ N 13°2′2.29″ E) and in the garden of the SAAF Department, and were used to infest the previously obtained potted plants. All infested plants were placed inside entomological cages (150 × 150 mesh, 160 µm aperture) in a climate-controlled room (25 ± 1 °C), with a RH of 70 ± 5% and a photoperiod of 16:8 h (L:D).

### 2.3. Rearing of Typhlodromus exhilaratus

Individuals of *T. exhilaratus* were collected in May 2023 from *Viburnum tinus* L. (Viburnaceae) plants growing in field (38°6′25.03″ N, 13°21′0.19″ E). The phytoseiid was reared on plexiglass plates measuring 100 × 100 × 4 mm as described by Tsolakis et al. [39], using a food mixture of pollen from *Oxalis pes-caprae* L. (Oxalidaceae), *Typha latifolia* L. (Typhaceae), and *Carpobrotus edulis* (L.) (Aizoacee). The rearing was maintained in a climate-controlled chamber with conditions at 25 ± 1 °C, 70 ± 5% RH, and a 16:8 h L:D photoperiod.

### 2.4. Adult Cohort of Aculops lycopersici

The *A. lycopersici* adults for the trials were obtained by transferring 200 individuals onto the abaxial surface of four *S. nigrum* leaves, placed in Petri dishes (Ø 150 mm, h 10 mm) on cotton wool saturated with distilled water. The adults were allowed to lay eggs for 24 h, afterwards removed. The presence of juvenile stages was monitored daily, and postembryonic development was tracked until adulthood. Since it was not possible to distinguish males from females under the stereomicroscope, a mixed population of both sexes (24 h old) was used in the trials.

### 2.5. Females and Eggs Cohort of Typhlodromus exhilaratus

To obtain eggs of the phytoseiid, the procedure described by Rizzo et al. [24] was adopted. Fifty females of *T. exhilaratus* were transferred from mass-breeding arenas to a new arena. After 24 h, females were removed and laid eggs (max 24 h old) were used for the experiments. The eggs were gently transferred into the experimental units using a fine brush (4/0).

To obtain fertilized females of *T. exhilarates* for the experiments, 50 females were taken from the colony and transferred to a new breeding arena. After 24 h, females were removed, leaving only the laid eggs. After about 8 days, the eggs developed into adults. Obtained females were kept for a further 48 h with males in 3:1 ratio (female:male) to allow mating.

### 2.6. Experimental Units for Aculops lycopersici and Typhlodromus exhilaratus

The EUs used in the toxicity tests on *A. lycopersici* consisted of leaf disks of *S. nigrum* (Ø 1.6 cm), while, for the tests on *T. exhilaratus*, leaf disks of bean [*Phaseolus vulgaris* L. (Fabaceae)] were used (Ø 3 cm). All EUs were placed with the abaxial surface facing upwards on wet cotton wool inside Petri dishes (Ø 100 mm, height 10 mm) to maintain their turgidity throughout the duration of the test.

### 2.7. Effects of Carlina Oxide and N-Alkylamides-Enriched Extract on Aculops lycopersici 

For each EU, five adults of *A. lycopersici* were transferred using a manual double-pin microdrill on which a human eyelash was attached to the end. This tool facilitated the transfer of the individuals, reducing the risk of damaging them given their small size.

Carlina oxide and the *N*-alkylamides-enriched extract were evaluated at six different concentrations (0, 320, 640, 1280, 2500, and 5000 µL L^−1^). Treatments with both products were carried out using a Potter Precision Spray Tower (Burkard Manufacturing Co. Limited, Woodcock Hill Industrial Estate, Rickmansworth, Hertfordshire WD3 1PJ, England), adopting different spray pressures depending on the properties of the product used. Carlina oxide, due to its chemical structure, is insoluble in water alone, and so it was diluted in acetone and sprayed at a pressure of 6.89 kPa, to avoid rapid evaporation of the solution and ensure uniform distribution over the experimental units. In contrast, the *N*-alkylamides-enriched extract, was diluted in water and acetone (4:1 ratio); therefore, a pressure of 62.05 kPa was applied. Each EU, for both products, was treated with 8 mL of solution at the specified concentration. The control units (concentration 0 µL L^−1^) were treated, respectively, with pure acetone in the case of the carlina oxide and with the water-acetone mixture in a 4:1 ratio in the case of the *N*-alkylamides-enriched extract. For each set of concentrations, ten repetitions were carried out (50 adults/concentration/extract).

To estimate the toxicity classes of the two extracts against the *A. lycopersici* population, the categories proposed by Hardman et al. [40] were adopted: (1) not toxic (mortality < 25%), (2) slightly toxic (mortality between 26% and 50%), (3) moderately toxic (mortality between 51% and 75%), and (4) very toxic (mortality > 76%). To obtain an accurate estimate of corrected mortality, it is necessary to exclude the natural mortality from the observed population. For this purpose, Abbott’s formula [41], which accounts for natural mortality, was applied.

### 2.8. Side Effects of Carlina Oxide and N-Alkylamides-Enriched Extract on Typhlodromus exhilaratus

Toxicity tests on the phytoseiid eggs and mated females were conducted at a concentration of 1280 µL L^−1^. This dose was chosen because it caused more than 90% of mortality on adults of *A. lycopersici*. The two products were mixed and applied following the same procedure as in Section 2.7. The control units (concentration 0 µL L^−1^) were treated, respectively, with pure acetone in the case of the carlina oxide and with the water-acetone mixture in a 4:1 ratio in the case of the *N*-alkylamides-enriched extract.

Toxicity tests on eggs were carried out treating five eggs placed on each EU. Egg hatching was observed daily for a period of three days after treatment, as this is the maximum embryonic development period for this species. Toxicity tests on the mated females were performed following the same procedure used for the phytoseiid eggs, transferring five females onto each EU. A mixture of pollen grains was placed on leaf disk as food once the sprayed surface was dry. Females were observed for four-day period after the treatment. In both tests, ten repetitions were carried out (50 eggs or 50 adults/concentration/extract).

The toxicity categories established by the International Organization for Biological Control for natural enemies, similar to those used for *A. lycopersici*, were applied to *T. exhilaratus*, considering Abbott’s corrected mortality. The categories are as follows: 1 = harmless (<30% mortality), 2 = slightly harmful (30–79% mortality), 3 = moderately harmful (80–99% mortality), and 4 = harmful (>99% mortality) [42].

### 2.9. Statistical Analysis

Mortality data, expressed as fractions, were transformed using an arcsine-square-root equation prior to the general linear model analysis (GLM). To confirm the assumption of normality, a residual analysis was performed on the data. In the model, the mortality was included as the response variable and “compound/extract” (carlina oxide and *N*-alkylamides-enriched extract), “concentration” (0, 320, 640, 1280, 2500, and 5000 µL L^−1^), and “time” (1, 2, 3, and 4 days) were the categorical covariates. The interactions between factors were also taken into account. The mean survival time data were transformed using the square-root + 0.5 function before performing the GLM analysis. When significant differences between treatments were detected, mean values were compared using Tukey’s HSD test (*p* = 0.05). Adjusted mortality, calculated using Abbott’s formula, was applied in the Probit analysis. The lethal concentrations corresponding to 10% (LC_10_), 30% (LC_30_), 50% (LC_50_), and 90% (LC_90_) mortality were estimated using the Probit model, with a 95% confidence interval. The number of dead mites was used as response in Event, the log-transformed concentrations as Stress (stimulus), and “compound/extract” as Factor, assuming the Weibull distribution and the maximum likelihood for estimation method. If the Pearson Goodness-of-fit test was significant, each “compound/extract” was analyzed singularly, eliminating the Factor from the model.

All statistical analyses were carried out using Minitab 19.0 software (Minitab Inc., State College, PA, USA).

## 3. Results

### 3.1. Analyses of Carlina Oxide and N-Alkylamides-Enriched Extract

Carlina oxide purity was determined as mentioned in Section 2.1 and resulted in 96.0%.

As regards to the *N*-alkylamides-enriched extract, the main target compounds were quantified by HPLC-DAD. The major compound was spilanthol (44.6 ± 0.2 g/100 g), while (*2E*,*6Z*,*8E*)-*N*-(2-methylbutyl)-2,6,8-decatrienamide (2.7 ± 0.0 g/100 g), (*2Z*)-*N*-isobutyl-2-nonene-6,8-diynamide, (*2E*)-*N*-(2-metilbutyl)-2-undecene-8,10-diynamide, and (*2E*)-*N*-isobutyl-2-undecene-8,10-diynamide were present in minor amounts (Table 1).

### 3.2. Toxicity of Carlina Oxide and N-Alkylamides-Enriched Extract on Aculops lycopersici

The overall mortality caused by the two plant-derived products against *A. lycopersici* was similar (*F*_1, 432_ = 3.77; *p* = 0.053), but it was significantly influenced by concentration (*F*_5, 432_ = 26.52; *p* < 0.001) and time (*F*_3, 432_ = 388.55; *p* < 0.001). The absence of significant interaction between *compound/extract* and *concentration* indicated that concentrations adopted have caused similar toxic effects to the eriophyid (*F*_5, 432_ = 1.74; *p* = 0.123). However, the interaction between *compound/extract* and *time* (*F*_3, 432_ = 9.59; *p* < 0.001), *concentrations* and *time* (*F*_15, 432_ = 39.31; *p* < 0.001), as well as that between the three factors (*F*_15, 432_ = 2.03; *p* = 0.013), showed significant differences, suggesting that the toxicity varied over the test period. Indeed, for all concentrations tested, the highest mortality was recorded within the first 24 h, and an additional mortality of less than 15% was registered in the following days (Table 2).

The highest concentrations of carlina oxide (5000 and 2500 µL L^−1^) showed a strong toxic effect on *A. lycopersici* adults after 24 h, with mortality rates of 100% (Table 2), whereas the *N*-alkylamides-enriched extract at the same concentrations achieved comparable mortality only after three days. Concentration of 1280 µL L^−1^ registered a similar trend of mortality during the test period, reaching the same level of mortality at the end of the tests (98.00 ± 2.00%). However, the two lower concentrations (640 and 320 µL L^−1^) showed significant differences between the two products (Table 2). Carlina oxide induced an approximately 1.8-fold higher mortality than the same concentrations of *N*-alkylamides-enriched extract (Table 2). Consequently, the two lower concentrations of *N*-alkylamides-enriched extract were classified as slightly toxic (class 2) according to the toxicity categories proposed by Hardman et al. [40], whereas the same concentrations of carlina oxide fall into the moderate (class 3) and high (class 4) toxicity classes, for 320 and 640 µL L^−1^, respectively (Table 2).

The GLM analysis of mean survival time revealed significant differences between the products (*F*_1, 588_ = 37.10; *p* < 0.001), concentrations (*F*_5, 588_ = 196.07; *p* < 0.001), and their interaction (*F*_5, 588_ = 11.55; *p* < 0.001). In both products, the three highest concentrations (5000, 2500, and 1280 μL L^−1^) resulted in a mean survival time of less than 24 h. In contrast, at lower concentrations (640 and 320 μL L^−1^), carlina oxide exerted a more pronounced toxic effect than the *N*-alkylamides-enriched extract, reducing mean survival time to under 48 h (Table 2). 

The LC_50_ for carlina oxide was 205.32 μL L^−1^ and the estimated probit data well fit in the linear model (χ^2^ = 0.14; *p* = 0.98) (Table 3).

At the same lethal concentration rank (LC_50_), a similar value (253.79 μL L^−1^) has been calculated for the *N*-alkylamides-enriched extract, but the Pearson goodness-of-fit test indicated that estimated data for this extract did not fit the linear model (χ^2^ = 32.53; *p* < 0.001). This discrepancy is likely due to the bimodal distribution of mortality rates: 98–100% mortality at the three highest concentrations (1280–5000 μL L^−1^) and 48–50% at the two lowest concentrations (640 and 320 μL L^−1^). Therefore, the LC_50_ value for the *N*-alkylamides-enriched extract should be considered indicative.

### 3.3. Toxicity of Carlina Oxide and N-Alkylamides-Enriched Extract on Typhlodromus exhilaratus

Statistical analysis on the percentage of hatched eggs/day and on the overall hatching rate showed significant differences between concentrations (0, 1280 μL L^−1^) (*F*_1, 110_ = 7.14; *p* = 0.009), time (1–3 days) (*F*_2, 110_ = 21.28; *p* < 0.001), and the three-way interaction (product × concentration × time) (*F*_2, 110_ = 23.98; *p* < 0.001).

Carlina oxide at 1280 μL L^−1^ negatively affected egg hatching of the phytoseiid, showing a reduction of approximately 43.18% compared to the same concentration of the *N*-alkylamides-enriched extract, and 50% compared to the control. A slight delay in hatching was also observed compared to the control: specifically, the highest number of hatched eggs in the *N*-alkylamides-enriched extract occurred on the third day of observation, while about 2/3 of the eggs hatched in the first day in the control (Table 4).

Concerning the effect on *T. exhilaratus* females, Carlina oxide and the *N*-alkylamides-enriched extract were found to range from harmless (class 1) to slightly harmful (class 2). Daily mortality showed statistically significant differences between compound/extract and control (*F*_1, 144_ = 4.57; *p* = 0.034), especially at the first day after spraying, concentrations (*F*_1, 144_ = 18.34; *p* < 0.001), and *time* (*F*_3, 144_ = 8.97; *p* < 0.001). The paired interactions extract × concentration and extract × time as well as the three-way interaction (extract × concentration, extract × time) were not significant (*F*_3, 144_ = 1.59; *p* = 0.194), indicating a similar effect of the two extracts on *T. exhilaratus* females. (Table 5).

On the other hand, the GLM analysis on mean survival time showed significant differences between the extracts (*F*_1196_ = 5.07; *p* = 0.025) and concentrations (*F*_1196_ = 25.81; *p* < 0.001), but not for the interaction between the two factors (*F*_1196_ = 2.35; *p* = 0.127). Again, carlina oxide demonstrated a stronger toxic effect: females treated with 1280 μL L^−1^ showed a mean survival time of 0.68 days shorter than those treated with the same concentration of the *N*-alkylamides-enriched extract, and 1.22 days shorter than the control group (Table 5).

Regarding oviposition rate of the phytoseiid, statistical analysis showed significant differences between compound/extract (*F*_1150_ = 69.36; *p* < 0.001), concentrations (*F*_1150_ = 44.81; *p* < 0.001), and their interaction (*F*_1150_ = 32.59; *p* < 0.001). Females exposed to carlina oxide exhibited, approximately, a five-fold reduction in oviposition rate compared to both the control and the *N*-alkylamides-enriched extract, while females treated with the *N*-alkylamides-enriched extract laid a number of eggs comparable to the control (Table 5).

## 4. Discussion

Carlina oxide is a polyacetylene belonging to the phytoalexins, a class of plant defense molecules. The purity of carlina oxide detected in this study was consistent with that reported in previous works [24]. Concerning the composition of the *N*-alkylamides-enriched extract of *A. oleracea*, its content in *N*-alkylamides was nearly identical with the work of Ferrati et al. [37].

In the present study, both tested products demonstrated high toxicity against *A. lycopersici*, with carlina oxide exhibiting slightly greater toxicity compared to the *N*-alkylamides-enriched extract. A mortality rate of 73% was obtained by Alhewairini [43], using a synthetic active ingredient (Oxamyl) at a dose of 480 µL L^−1^. This result is particularly relevant when compared to our findings, where carlina oxide caused 70% mortality at a lower concentration of 320 µL L^−1^. The efficacy of carlina oxide was also evident in terms of speed of action: in our bioassay, the highest tested concentration (5000 µL L^−1^) caused 100% mortality of all treated individuals within 24 h, aligning with the observations by Rizzo et al. [24], who reported 100% mortality in *Tetranychus urticae* Koch females after 48 h at the same concentration. Similarly, Novák et al. [44] recorded 96.7% mortality in adult *Metopolophium dirhodum* (Walker) at a dose of 3000 µL L^−1^, while Tortorici et al. [45] showed that carlina oxide exerts both toxic and repellent effects on adult *Philaenus spumarius* (L.), achieving 90% mortality after 72 h using a 3% nanoemulsion. Carlina oxide and related formulations have been tested against a variety of target insects, such as disease vectors, agricultural pests, and stored-product insects, showing good to excellent results [24,46,47,48,49]. Its mode of action is still unknown, but it probably relies on the triple bond of the propynyl chain that produces radicals generating oxidative damage [50]. This phenomenon could also be enhanced by exposure to UV light [21]. Another possible mechanism could be based on the interaction with the insect γ-aminobutyric acid (GABA) receptor [50] or on the inhibition of the acetylcholinesterase enzyme [34].

As for the acaricidal activity of the N-alkylamides-enriched extract, our study recorded 100% mortality in all tested *A. lycopersici* individuals after 72 h following treatment with a 5000 µL L^−1^ dose. Moreno et al. [51], using a hexane extract from the aerial parts of *A. oleracea*, reported 100% mortality of *Tuta absoluta* (Meyrick) (Lepidoptera: Gelechiidae) larvae after 6 h of topical exposure at a dose of 10 µg of extract per mg of insect body weight. Additionally, Gouvêa et al. [52] tested ethanolic and aqueous extracts of *A. oleracea*, against the aphids *Myzus persicae* Sulzer and *Lipaphis erysimi* (Kalt.) (Hemiptera: Aphididae). The ethanolic extract induced 90% mortality at a concentration of 10 g L^−1^, while the aqueous one showed lower efficacy. Extracts obtained from *A. oleracea* have also demonstrated promising insecticidal activity against a wide range of harmful insect species, including members of Blattodea and Lepidoptera [51,52,53]. The extract herein employed is primarily composed of N-alkylamides (62.2%), with the main components being spilanthol (44.6%) and N-(2-methyl-butyl)-2,6,8-decatrienamide (2.7%). Spilanthol is known for its high penetration capacity in insects and, although its mode of action is not fully understood, it appears to affect the central nervous system [35]. This hypothesis is supported by studies on the hard tick *Rhipicephalus microplus* (Canestrini) (Ixodida: Ixodidae), in which extracts obtained from *A. oleracea* with various spilanthol concentrations were tested to assess the relationship between acaricidal efficacy and the concentration of the active compound [54].

Finally, the results of the present study are comparable to those reported by Giordano et al. [25], who evaluated the efficacy of three EOs obtained from species belonging to the Lamiaceae family against *A. lycopersici*. Among the tested EOs, the *Origanum vulgare* L. EO was the most effective, causing 90% mortality four days after treatment.

Regarding the side effects on natural enemies, Rizzo et al. [24] observed very low mortality in *Neoseiulus californicus* (McGregor) females when exposed to a concentration of 5000 µL L^−1^ of carlina oxide, while a concentration of 1280 µL L^−1^ had no effect on egg hatching. However, these authors reported a strong repellent effect when the females were directly sprayed and remained on the treated leaf disk. Our results contrast with these findings: at the concentration of 1280 µL L^−1^ we observed a toxic but non-repellent effect on *T. exhilaratus* females and a moderate negative impact on egg hatching. These differences may be explained by varying detoxification capabilities between the two phytoseiids species, as well as by potential mutations in the pesticide target site that could influence their response to the compound, as suggested by Duso et al. [55]. In contrast, the toxic effect of the *N*-alkylamides-enriched extract on females and eggs of the phytoseiid was much more limited, demonstrating the potential use of this natural extract in IPM programs.

Our study suggests that both carlina oxide and the *N*-alkylamides-enriched extract represent promising natural alternatives to synthetic acaricides, demonstrating high efficacy in controlling *A. lycopersici*. However, laboratory studies are the first step in evaluating plant products, but some gaps must be filled before they can be commercially adopted. Generally, botanical products are susceptible to oxidative deterioration when exposed to air, sun light (UV-radiation), and heat, making the use of a carrier mandatory before their use in field trials [56]. Encapsulation of EOs, plant extracts, or pure compunds in appropriate carriers is the most promising technique where the bioactive substance is entrapped and protected by a carrier, which allows one to control the rate of bioactive release [57]. Indeed, controlled release technology aims at effective pesticide treatment, avoidance of side effects, prolonging efficiency, and boosting safety and reliability [58]. Consequently, further research is needed to optimize formulation and application strategies to maximize the effectiveness of these natural products under field conditions.

## Figures and Tables

**Table 1:** *N*-alkylamides concentration in the *N*-alkylamides-enriched extract, as quantified by HPLC-DAD.

*N*-Alkylamides	Concentration (g/100 g) ± SD ^a^
(*2Z*)-*N*-isobutyl-2-nonene-6,8-diynamide	0.6 ± 0.0
(*2E*)-*N*-isobutyl-2-undecene-8,10-diynamide	0.3 ± 0.0
(*2E,6Z,8E*)-*N*-isobutyl-2,6,8-decatrienamide (spilanthol)	44.6 ± 0.2
(*2E,7Z*)-*N*-isobutyl-2,7-decadienamide	0.4 ± 0.0
(*2E*)-*N*-(2-metilbutyl)-2-undecene-8,10-diynamide	
(*2E,6Z,8E*)-*N*-(2-metilbutyl)-2,6,8-decatrienamide	2.7 ± 0.0
Total *N*-alkylamides	48.6 ± 0.2

^a^ Average concentration of *N*-alkylamides found in the *N*-alkylamide-enriched extract as the mean of two independent analysis ± standard deviation.

**Table 2:** Susceptibility of adults of *Aculops lycopersici* to various concentrations of carlina oxide and *N*-alkylamides-enriched extract.

Compound/Extract	Concentration	Mortality (%) (Mean ± S.E.)				Survival Time Days	Overall Mortality	Adjusted Mortality (Abbott)	Toxicity Class *
	(µL L^−1^)	Day 1	Day 2	Day 3	Day 4	(mean ± SE)	(% ± SE)	(%)	(-)
Carlina oxide	5000	100.00 ± 0.00 a	-	-	-	0.00 ± 0.00 a	100.00 ± 0.00 a	100.00	4
	2500	100.00 ± 0.00 a	-	-	-	0.00 ± 0.00 a	100.00 ± 0.00 a	100.00	4
	1280	88.00 ± 4.42 ab	8.00 ± 3.27 f	2.00 ± 2.00 f	0.00 ± 0.00 f	0.20 ± 0.10 ab	98.00 ± 2.00 a	97.78	4
	640	66.00 ± 8.97 cd	10.00 ± 4.47 f	14.00 ± 4.27 f	0.00 ± 0.00 f	0.78 ± 0.18 b	90.00 ± 4.47 a	88.89	4
	320	44.00 ± 8.33 de	14.00 ± 6.70 f	4.00 ± 2.67 f	8.00 ± 3.27 f	1.66 ± 0.25 c	70.00 ± 5.37 b	66.67	3
	0	0.00 ± 0.00 f	6.00 ± 3.06 f	4.00 ± 2.67 f	0.00 ± 0.00 f	3.74 ± 0.11 e	10.00 ± 3.33 c	0.00	-
*N*-alkylamides-enriched extract	5000	94.00 ± 3.06 ab	4.00 ± 2.67 f	2.00 ± 2.00 f	-	0.08 ± 0.05 a	100.00 ± 0.00 a	100.00	4
	2500	92.00 ± 4.42 ab	6.00 ± 4.27 f	2.00 ± 2.00 f	-	0.10 ± 0.05 a	100.00 ± 0.00 a	100.00	4
	1280	78.00 ± 7.57 bc	12.00 ± 3.27 f	6.00 ± 6.00 f	2.00 ± 2.00 f	0.38 ± 0.12 ab	98.00 ± 2.00 a	97.78	4
	640	22.00 ± 9.64 ef	10.00 ± 6.15 f	10.00 ± 6.15 f	8.00 ± 4.42 f	2.54 ± 0.24 d	50.00 ± 6.15 b	44.44	2
	320	18.00 ± 8.14 ef	10.00 ± 8.03 f	10.00 ± 4.47 f	10.00 ± 5.37 f	2.68 ± 0.23 d	48.00 ± 6.80 b	42.22	2
	0	4.00 ± 2.67 f	6.00 ± 3.06 f	0.00 ± 0.00 f	0.00 ± 0.00 f	3.66 ± 0.15 e	10.00 ± 3.33 c	0.00	-

Different letters indicate significant differences among extracts and concentrations for daily mortality, survival time, and overall mortality. Tukey’s multiple comparison tests (*p* < 0.05) were applied after GLM analysis. * Toxicity class: 1—not toxic (<25%); 2—slight toxic (26–50%); 3—moderately toxic (51–75%); and 4—very toxic (>76%) after Hardman et al. [40].

**Table 3:** Lethal concentrations of carlina oxide and *N*-alkylamides-enriched extract against adults of *Aculops lycopersici*.

Compound/Extract	LC_10_ µL L^−1^ (95% CI)	LC_30_ µL L^−1^ (95% CI)	LC_50_ µL L^−1^ (95% CI)	LC_90_ µL L^−1^ (95% CI)	LC_95_ µL L^−1^ (95% CI)	Intercept ± SE	Slope ± SE	Goodness of Fit *χ*^2^ (d.f.)
Carlina oxide	67.40 (13.22–127.83)	130.16 (42.92–204.40)	205.32 (95.90–286.23)	625.48 (494.02–902.00)	857.76 (653.49–1502.62)	−6.12 ± 1.63	2.64 ± 0.60	0.14 (3) *p* = 0.98
*N*-alkylamides-enriched extract	39.00 (24.22–55.68)	117.93 (87.44–150.71)	253.79 (202.28–315.89)	1651.58 (1192.94–2537.64)	2808.66 (1905.10–4743.84)	−3.78 ± 0.34	1.57 ± 0.13	32.53 (6) *p* < 0.001

Tukey’s multiple comparison tests (*p* < 0.05) were applied after GLM analysis.

**Table 4:** Side effects of carlina oxide and *N*-alkylamides-enriched extract on *Typhlodromus exhilaratus* eggs.

Compound/Extract	Concentration	Hatching/Day (%) Mean ± SE			Overall Hatching
	(µL L^−1^)	Day 1	Day 2	Day 3	(% ± SE)
Carlina oxide	1280	44.00 ± 4.99 ab	4.00 ± 2.67 a	2.00 ± 2.00 a	50.00 ± 4.47 a
	0	40.00 ± 9.89 b	38.00 ± 9.17 b	22.00 ± 3.59 b	100.00 ± 0.00 b
*N*-alkylamides-enriched extract	1280	22.00 ± 7.57 b	28.00 ± 8.00 b	38.00 ± 8.14 b	88.00 ± 6.11 c
	0	68.00 ± 7.42 a	32.00 ± 7.42 b	0.00 a	100.00 ± 0.00 b

Different letters in the column indicate significant differences among compound/extract and concentrations. Tukey’s multiple comparison tests (*p* < 0.05) were applied after GLM analysis.

**Table 5:** Mortality of *Typhlodormus exhilaratus* females treated with carlina oxide and *N*-alkylamides-enriched extract.

Compound/Extract	Concentration	Mortality/Day (%, Mean ± SE)				Overall Mortality	Survival Time Days	Fecundity Eggs/Female/Day	Adjusted Mortality Abbott	Toxicity Class *
	(µL L^-1^)	Day 1	Day 2	Day 3	Day 4	(% ± SE)	(mean ± SE)	(mean ± SE)	(%)	(-)
**Carlina oxide**	1280	28.00 ± 8.00 a	8.00 ± 3.27 b	2.00 ± 2.00 b	6.00 ± 4.27 b	44.00 ± 8.84 a	2.54 ± 0.26 a	0.11 ± 0.03 a	39.13	2
	0	2.00 ± 2.00 b	4.00 ± 2.67 b	2.00 ± 2.00 b	0.00 ± 0.00 b	8.00 ± 3.27 b	3.76 ± 0.12 b	0.48 ± 0.04 b	0.00	-
***N-*** **alkylamides-enriched extract**	1280	12.00 ± 5.33 a	10.00 ± 4.47 b	0.00 ± 0.00 b	0.00 ± 0.00 b	22.00 ± 4.67 c	3.22 ± 0.21 b	0.59 ± 0.05 b	18.75	1
	0	2.00 ± 2.00 b	2.00 ± 2.00 b	0.00 ± 0.00 b	0.00 ± 0.00 b	4.00 ± 2.67 b	3.86 ± 0.10 b	0.58 ± 0.03 b	0.00	-

Different letters indicate significant differences among extracts and concentrations. Tukey’s multiple comparison tests (*p* < 0.05) were applied after GLM analysis. * Toxicity classes were defined on corrected mortality after Sterk et al. [42].

## Data Availability

The data presented in this study are available on request from the corresponding author.
